# Case Report: Upadacitinib treatment for Behçet’s syndrome with intestinal damage

**DOI:** 10.3389/fmed.2026.1808317

**Published:** 2026-04-17

**Authors:** Ling Yuan, Yingying Sun, Jianjun Yan

**Affiliations:** Department of Dermatology, Shandong Provincial Hospital Affiliated to Shandong First Medical University, Jinan, Shandong, China

**Keywords:** Behçet’s syndrome, intestinal damage, JAK inhibitor, small molecule drug, upadacitinib

## Abstract

We report the case of a 17-year-old male presenting with recurrent, debilitating oral and genital ulcerations. Diagnostic evaluation confirmed multisystemic Behçet’s syndrome with multifocal intestinal involvement. Following the exclusion of infectious contraindications, the patient was initiated on upadacitinib (15 mg daily) as a primary systemic intervention. Remarkable clinical remission of mucocutaneous and gastrointestinal ulcerations was achieved within 1 month. Sustained improvement was observed during subsequent follow-up, suggesting that upadacitinib may serve as an effective therapy for multisystemic Behçet’s syndrome in adolescent patients.

## Introduction

Behçet’s syndrome (BS) is a complex multisystem inflammatory disease which affects the skin, mucosa, joints, eyes, gastrointestinal tract, vascular system, and central nervous system (1). While the etiology of this multisystemic disorder involves a complex interplay of infectious, genetic, epigenetic, and immunological factors, its precise pathogenesis remains elusive (2).

Currently, the treatment strategies for BS include topical anti-inflammatory therapy, colchicine, glucocorticoids, and synthetic or biologic immunosuppressive agents (1). Previous studies have demonstrated the activation of the Janus kinase-signal transducer and activator of transcription (JAK-STAT) signaling pathway in BS, suggesting that JAK inhibitors may be beneficial (3). Here, we report a case of BS with intestinal involvement that showed a favorable response to upadacitinib, an oral selective JAK1 inhibitor.

**Figure 1:**
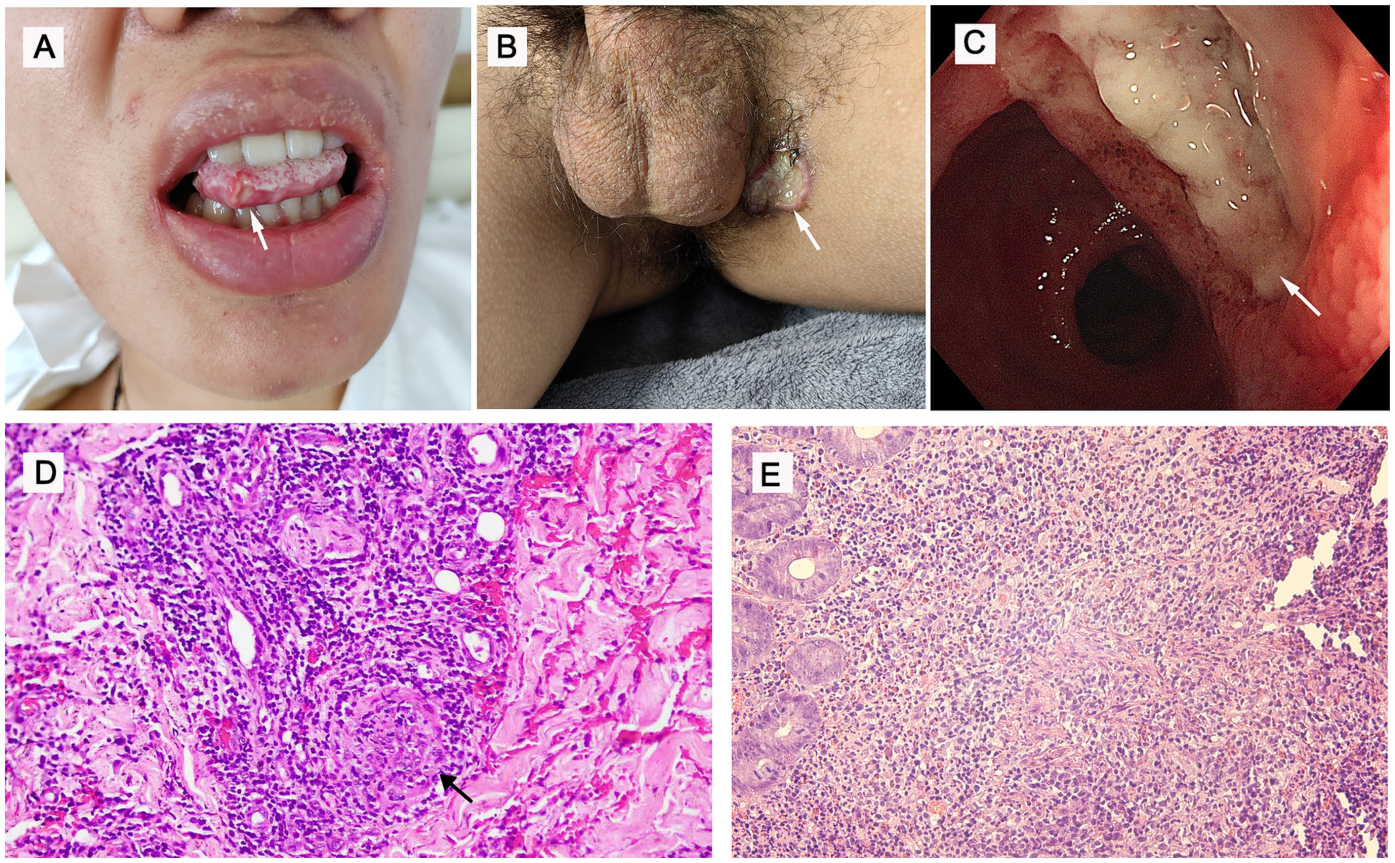
Clinical manifestations, ileocolonoscopy, and pathological examination of Behçet’s syndrome. **(A,B)** Deep ulcers in the tongue and left inguinal region (arrow). **(C)** Ulcer in the terminal ileum (approximately 0.6 × 0.6 cm, arrow). **(D)** Histological examination of the ulcers in the left inguinal (×10). **(E)** Histological examination of the ulcers in the histological examination of the ulcers in the intestinal mucosa (×20). Response of Behçet’s syndrome to upadacitinib.

**Figure 2:**
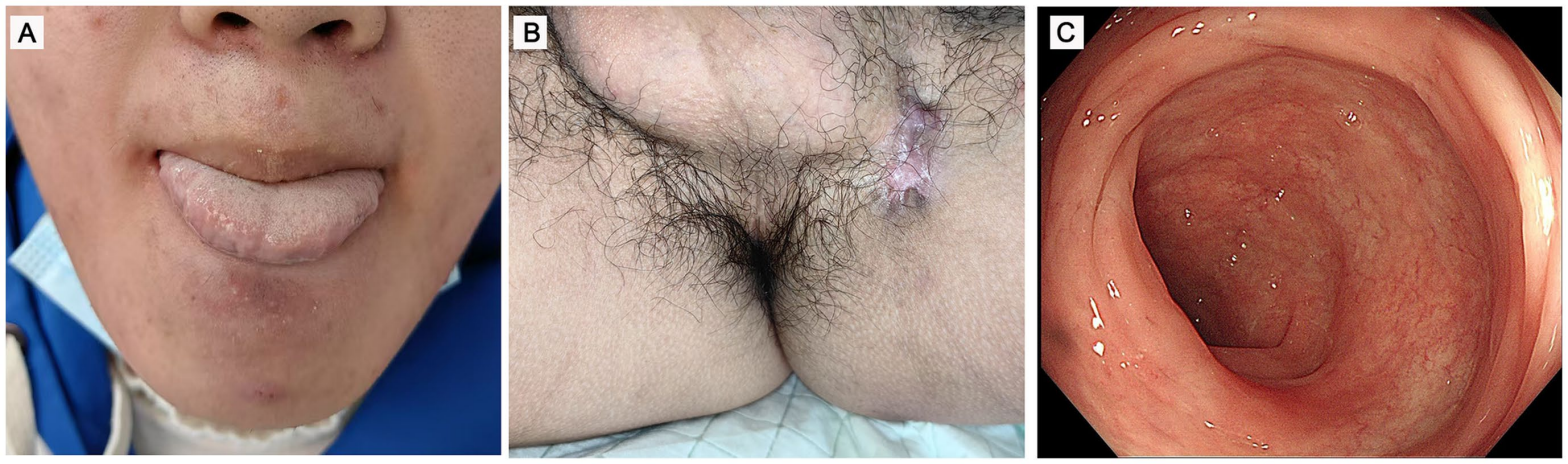
After treatment with upadacitinib, the deep ulcers in the tongue **(A)**, left inguinal region **(B)**, and intestinal mucosa **(C)** have healed.

We report the case of a 17-year-old boy presented with a 7-year history of recurrent oral and genital mucosal ulcers, involving the left inguinal region and experiencing abdominal pain for 2 months. The physical examination revealed a deep, painful ulcer with an erythematous halo on the lateral border of the tongue. Simultaneously, a well-circumscribed, punched-out ulcer with a fibrinous base was noted in the left inguinal fold. The scrotum exhibited post-inflammatory hyperpigmentation, sequelae of recurrent prior ulcerations (Figures 1A,B). Review of systems was unremarkable, with the patient denying fever, headache, arthralgia, pseudofolliculitis, and blurred vision; subsequent ophthalmic examination confirmed no abnormalities. Upon admission, comprehensive laboratory evaluations revealed significant systemic inflammation, characterized by an elevated C-reactive protein (CRP) level of 85.44 mg/L (normal <5 mg/L) and an accelerated erythrocyte sedimentation rate (ESR) of 46 mm/h. Bacterial cultures from the ulcer surfaces and screening for infectious etiologies—including CMV, EBV, HSV, T-SPOT. TB assay, chest computed tomography (CT), hepatitis B/C, syphilis, and HIV—were all negative (Supplementary Table 1). Due to persistent abdominal pain, ileocolonoscopy was performed, revealing a terminal ileal ulcer (approximately 0.6 × 0.6 cm) and multiple colonic ulcers (Figure 1C). Histopathological examination (hematoxylin and eosin staining) of the left inguinal lesion revealed a mixed inflammatory infiltrate predominantly composed of lymphocytes with scattered neutrophils (Figure 1D). Similarly, intestinal mucosal biopsy demonstrated moderate-to-severe chronic active inflammation and ulceration, with marked interstitial congestion and edema (Figure 1E). Although lymphocytic vasculitis is not pathognomonic for BS, the pathological findings were considered consistent with the clinical context of chronic inguinal ulceration. Based on the International Study Group (ISG) criteria, the patient fulfilled the mandatory criterion of recurrent oral ulceration, together with recurrent genital ulceration and cutaneous lesions, thereby meeting the diagnostic criteria for BS (4). In the differential diagnosis, Crohn’s disease was primarily considered due to the gastrointestinal involvement; however, the presence of recurrent oral and genital ulcers strongly favored BS. Furthermore, the discrete round ulcers covered with white exudate observed in this patient differed morphologically from the longitudinal ulcers typical of Crohn’s disease. Systemic lupus erythematosus (SLE) was also excluded, as the patient’s oral ulcers were painful and comprehensive immunological workup (including ANA and anti-dsDNA) yielded no supportive evidence. To further evaluate potential intestinal or vascular involvement, computed tomography enterography (CTE) was recommended; however, the patient’s guardians declined the procedure. After establishing the diagnosis, several treatment options were discussed with the patient and family. Systemic glucocorticoid therapy was initially proposed; however, it was declined by the patient’s guardians due to concerns regarding the potential long-term adverse effects of corticosteroids. Given the patient’s significant abdominal pain and multiple intestinal ulcers, as well as the potential gastrointestinal side effects of glucocorticoids and colchicine, we adopted a cautious approach in treatment selection. Adalimumab was declined due to potential non-adherence to subcutaneous injections. The oral medication was selected as a more acceptable and manageable therapeutic alternative for the patient. Due to its greater JAK1 selectivity and the clinical convenience of its dosing regimen compared to tofacitinib, upadacitinib was initiated as the preferred oral therapeutic agent. Therefore, the patient was treated with upadacitinib (15 mg/d). One month later, the ulcers in the tongue and left inguinal region healed, and the abdominal pain disappeared (Figures 2A,B). After 3 months, we did the ileocolonoscopy again and found that the multiple ulcers of the intestine were healed (Figure 2C).

Case reports and small case series have indicated that JAK inhibitors may be effective in the management of Behçet syndrome, particularly when used in combination with glucocorticoids and other immunosuppressive agents (5). However, evidence regarding JAK inhibitor monotherapy remains limited, particularly in treating BS with intestinal involvement. Moreover, to the best of our knowledge, this is one of the few reports evaluating upadacitinib monotherapy for addressing intestinal BS.

Proinflammatory cytokines and increased JAK activation are involved in BS (3). Given the shared inflammatory pathways, upadacitinib has been investigated in other immune-mediated conditions; for instance, it has shown therapeutic potential in inflammatory bowel disease (IBD), providing a mechanistic rationale for its use in intestinal inflammatory disorders (6). This mechanistic overlap provides a clinical rationale for exploring JAK1 inhibition in BS patients with gastrointestinal involvement. Furthermore, emerging clinical evidence suggests that upadacitinib, when combined with glucocorticoids or other immunosuppressive agents, shows good therapeutic efficacy in BS and may significantly reduce glucocorticoid requirements (7). The efficacy and safety profile of upadacitinib for treating BS with intestinal involvement must be further verified by prospective clinical trials with large sample sizes. In addition, more comprehensive investigations are necessary to elucidate the underlying mechanisms of upadacitinib in this context.

In summary, we report a case of BS with significant intestinal involvement that achieved a favorable clinical response following upadacitinib monotherapy. Given the patient’s refusal of systemic glucocorticoids and preference for an oral therapeutic agent over injectable biologics, upadacitinib was selected as a tailored treatment option based on its enhanced JAK1 selectivity and the underlying role of the JAK–STAT pathway in BS pathogenesis. This case suggests that JAK1 inhibitors may represent a promising oral alternative for refractory or treatment-selective cases. Nonetheless, prospective clinical trials with larger cohorts and extended follow-up durations are essential to fully validate its long-term safety and establish its place in the standard management of BS.

## Data Availability

The original contributions presented in the study are included in the article/Supplementary material, further inquiries can be directed to the corresponding author.

## Supplementary material

SUPPLEMENTARY TABLE 1 Baseline clinical laboratory and serological parameters.
