# Supplementary material for: Case Report: Upadacitinib treatment for Behçet’s syndrome with intestinal damage
Source: Front Med (Lausanne). 2026 Apr 17;13:1808317. doi: 10.3389/fmed.2026.1808317 (PMC13132688; doi:10.3389/fmed.2026.1808317)
Supplement: SUPPLEMENTARY TABLE 1 — Baseline clinical laboratory and serological parameters. [file Table_1.docx]

| **Category** | **Parameter** | **Value** |
| --- | --- | --- |
| **Complete Blood Count** | White blood cell count | 11.46 ×10⁹/L |
|  | Red blood cell count | 4.98 ×10¹²/L |
|  | Hemoglobin | 132 g/L |
|  | Platelet count | 338 ×10⁹/L |
| **Inflammatory Markers** | C-reactive protein (CRP) | 85.44 mg/L |
|  | Serum amyloid A (SAA) | 266.58 mg/L |
|  | Erythrocyte sedimentation rate (ESR) | 46 mm/h |
|  | Procalcitonin (PCT) | 0.07 ng/mL |
|  | Interleukin-6 (IL-6) | 9.53 pg/mL |
| **Coagulation Parameters** | D-dimer | 0.35 mg/L |
|  | Prothrombin time (PT) | 13.90 s |
|  | Prothrombin activity | 70.00% |
|  | International normalized ratio (INR) | 1.25 |
|  | Prothrombin ratio | 1.24 |
|  | Activated partial thromboplastin time (aPTT) | 31.60 s |
|  | aPTT ratio | 1.01 |
|  | Thrombin time (TT) | 13.20 s |
|  | Fibrinogen | 4.05 g/L |
| **Liver Function Tests** | Aspartate aminotransferase (AST) | 11 U/L |
|  | Alanine aminotransferase (ALT) | 7 U/L |
|  | Gamma-glutamyl transferase (GGT) | 33 U/L |
|  | Alkaline phosphatase (ALP) | 108 U/L |
|  | Glutamate dehydrogenase (GLDH) | 1.4 U/L |
|  | Adenosine deaminase (ADA) | 20.70 U/L |
|  | Total bile acids | 6.00 μmol/L |
|  | Glycocholic acid | 1.89 μg/mL |
|  | Prealbumin | 93.00 mg/L |
|  | Total protein | 80.0 g/L |
|  | Albumin | 37.0 g/L |
| **Biochemical Parameters** | Glucose | 4.93 mmol/L |
|  | β-hydroxybutyrate | 0.349 mmol/L |
|  | Urea | 3.7 mmol/L |
|  | Creatinine | 68.70 μmol/L |
|  | eGFR | 133.21 mL/min/1.73 m² |
|  | Urea/creatinine ratio | 53.86 |
|  | Uric acid | 453 μmol/L |
|  | Cystatin C | 0.96 mg/L |
|  | β2-microglobulin | 2.18 mg/L |
|  | Retinol-binding protein | 17.60 mg/L |
|  | Complement C1q | 187.90 mg/L |
|  | NGAL | 140.60 ng/mL |
| **Electrolytes and Metabolic Panel** | Calcium | 2.23 mmol/L |
|  | Phosphate | 1.57 mmol/L |
|  | Magnesium | 0.94 mmol/L |
|  | Potassium | 3.84 mmol/L |
|  | Sodium | 135.7 mmol/L |
|  | Chloride | 100.9 mmol/L |
|  | Carbon dioxide (CO₂) | 23.5 mmol/L |
|  | Osmolality | 270.03 mOsm/L |
|  | Anion gap | 15.14 mmol/L |
|  | Lactate dehydrogenase (LDH) | 181.00 U/L |
| **Immunological Tests** | Immunoglobulin A (IgA) | 6.33 g/L |
|  | Immunoglobulin M (IgM) | 0.57 g/L |
|  | Immunoglobulin E (IgE) | 146.00 IU/mL |
|  | Immunoglobulin G (IgG) | 22.00 g/L |
|  | Immunoglobulin G4 (IgG4) | 1620 mg/L |
|  | Complement C3 | 1.35 g/L |
|  | Complement C4 | 0.32 g/L |
| **Autoimmune Tests** | HLA-B27 | Negative |
|  | ANCA | Negative |
|  | ANA | Negative |
|  |  |  |
| **Hepatitis B Virus** | HBsAg | 0.00 IU/mL (Negative) |
|  | HBsAb | 66.49 mIU/mL (Positive) |
|  | HBeAg | 0.32 COI (Negative) |
|  | HBeAb | 2.01 COI (Positive) |
|  | HBcAb | 0.30 COI (Negative) |
| **Hepatitis C Virus** | Anti-HCV | 0.13 COI (Negative) |
| **Human Immunodeficiency Virus** | HIV Ag/Ab | 0.35 S/CO (Negative) |
| **Syphilis** | Treponema pallidum antibody | 0.09 S/CO (Negative) |
| **Herpesvirus Panel** | EBV DNA | <5000 copies/mL (Negative) |
|  | CMV DNA | <500 copies/mL (Negative) |
|  | HSV-1 DNA | Negative (-) |
|  | HSV-2 DNA | Negative (-) |
| **Tuberculosis Screening** | TB γ(N) | 33.91 |
|  | TB γ(T) | 47.61 |
|  | TB γ(P) | 5000.00 |
|  | Interferon-γ | 13.70 pg/mL (Negative) |
| **Clostridioides difficile** | Toxin B gene | Negative |
|  | tcdC (nt117 deletion) | Negative |
|  | Binary toxin | Negative |

| **Category** | **Parameter** | **Value** |
| --- | --- | --- |
| **Hepatitis B Virus** | HBsAg | 0.00 IU/mL (Negative) |
|  | HBsAb | 66.49 mIU/mL (Positive) |
|  | HBeAg | 0.32 COI (Negative) |
|  | HBeAb | 2.01 COI (Positive) |
|  | HBcAb | 0.30 COI (Negative) |
| **Hepatitis C Virus** | Anti-HCV | 0.13 COI (Negative) |
| **Human Immunodeficiency Virus** | HIV Ag/Ab | 0.35 S/CO (Negative) |
| **Syphilis** | Treponema pallidum antibody | 0.09 S/CO (Negative) |
| **Herpesvirus Panel** | EBV DNA | <5000 copies/mL (Negative) |
|  | CMV DNA | <500 copies/mL (Negative) |
|  | HSV-1 DNA | Negative (-) |
|  | HSV-2 DNA | Negative (-) |
| **Tuberculosis Screening** | TB γ(N) | 33.91 |
|  | TB γ(T) | 47.61 |
|  | TB γ(P) | 5000.00 |
|  | Interferon-γ | 13.70 pg/mL (Negative) |
| **Clostridioides difficile** | Toxin B gene | Negative |
|  | tcdC (nt117 deletion) | Negative |
|  | Binary toxin | Negative |
